# A descriptive analysis of multi-jurisdictional enteric illness messaging web analytics data, 2020–2022

**DOI:** 10.14745/ccdr.v52i04a03

**Published:** 2026-04-30

**Authors:** Vayshali Patel, Jennifer McWhirter, Melissa MacKay, Leslie Cheng, Melissa Phypers, Andrew Papadopoulos, Lauren Grant

**Affiliations:** 1 Department of Population Medicine, University of Guelph, Guelph, ON; 2 Centre for Food-borne, Environmental, and Zoonotic Infectious Diseases, Public Health Agency of Canada, Guelph, ON

**Keywords:** online communication, user experience, health communication, risk communication, enteric illness

## Abstract

**Background:**

Effective online communication is important for disseminating information during multi-jurisdictional enteric illness outbreaks in Canada. The Public Health Agency of Canada (PHAC) uses web-based Public Health Notices (PHNs) to communicate outbreak information and prevention measures. Despite this communication’s importance, no study has examined online engagement with PHAC’s PHNs.

**Objective:**

To assess access to and engagement with online information on multi-jurisdictional enteric illness outbreaks by analyzing website traffic and engagement metrics for PHAC’s PHNs.

**Methods:**

Data on page and screen metrics, geographic location, device and browser types, and traffic source metrics for PHAC’s PHN webpages (2020–2022) were obtained. Descriptive statistics were calculated for page and screen metrics data. Proportional frequencies were calculated for geographic location, device type, and traffic source metrics. Data were tabulated and visualized using R Studio.

**Results:**

Public Health Notice webpages had an average of 2,729±16,685 page views and 2,490±15,201 visits; decreasing (but not significantly) over the study period. Average session duration was 165±124 seconds; increasing (but not significantly) over time. Most visits originated in Canada (89.0%±4.2%) and were from mobile devices (74.6%±3.3%). Traffic sources were primarily search (49.1%±13.0%), followed by direct (23.9%±6.7%), social media (21.2%±8.4%), and referral (5.7%±2.5%). The geographic location, device type, and traffic source changed significantly year by year.

**Conclusion:**

Engagement with PHAC’s PHN webpages declined over the three years of the study, while mobile and search-driven access dominated and levels remained consistent over time. Social media generated comparatively little traffic. These findings suggest opportunities to enhance search optimization and social media amplification to improve outbreak communication.

## Introduction

As of January 2024, nearly 37 million Canadians were Internet users ((1)). With two-thirds of Canadians searching for health information online ((2)), the Internet has increasingly become a source of such information ((3–5)), including for topics such as disease diagnosis ((6)), decisions about seeking medical care ((7)), and updates on current events, such as outbreaks ((8)). This makes the Internet a valuable communication tool for information dissemination, including during multi-jurisdictional enteric illness outbreaks.

Enteric illnesses, primarily caused by pathogens affecting the gastrointestinal system, are a serious concern in Canada ((9)). Each year, there are an estimated four million cases of foodborne illness, leading to over 11,000 hospitalizations and 200 deaths ((9)). The Public Health Agency of Canada (PHAC) is responsible for communicating during multi-jurisdictional enteric illness outbreaks in Canada, defined as when two or more persons become ill from a common source and reside in two or more provinces/territories or Canada and another country. During an outbreak, public health authorities are expected to communicate timely and accurate information to the public ((10)). Public Health Agency of Canada uses Public Health Notices (PHNs), posted to the Government of Canada’s website, to share outbreak and health protection information with Canadians. These online information notices include key outbreak investigation details such as the number of cases per province/territory, the timeline, and health protection information. As new information becomes available, PHNs are updated accordingly to ideally prevent additional enteric illness.

Reputable organizations, such as government agencies, are often trusted as reliable sources of online information ((11–14)). This trust is a key component of credibility ((15)), which, in turn, influences how the public accepts and uses health information ((16)). If these organizations fail to develop user-friendly and credible websites, it can negatively impact users’ attitudes, lower their satisfaction, and increase concerns about the reliability of the information presented ((17)). To address these issues and enhance user experience, website traffic and engagement metrics offer valuable insights that are accurate, objective, and real-time, enabling organizations to optimize website performance and better meet user needs ((18)). In the context of public health communication, meeting user needs translates to ensuring the public can access, understand, and use health information, including in the context of multi-jurisdictional enteric illness outbreaks. Understanding web analytics for PHNs may offer clues for how well PHNs are meeting the health information needs of Canadians.

Previous studies have used website analytics to examine user behaviours on government websites ((19–21)). For instance, Begany *et al*. ((20)) investigated the relationship between website traffic and engagement with demographic characteristics in the context of open government data in the United States (US). Cheng and Chen ((21)) analyzed user behaviour on government websites during the COVID-19 pandemic, utilizing website traffic, engagement data, and focus groups to explore changes in engagement over time, as well as the impact of website characteristics and user experience. Tian *et al.* ((19)) used website traffic and engagement metrics to measure awareness of a specific health condition on the Centers for Disease Control and Prevention’s (CDC) website; however, no previous study has used web analytics data to describe government website use for multi-jurisdictional enteric illness outbreaks in Canada. The following engagement metrics were used because they are consistently reported in website analytics literature, and together, provide an overall view of how visitors access and engage with PHN webpages. This study uses website traffic and engagement data to quantify 1) page and screen metrics, 2) geographic location visits, 3) device and browser metrics, and 4) traffic sources for PHAC’s PHN webpages on the Government of Canada’s website over a three-year study period (2020–2022).

## Methods

### Data collection

**Figure 1:**
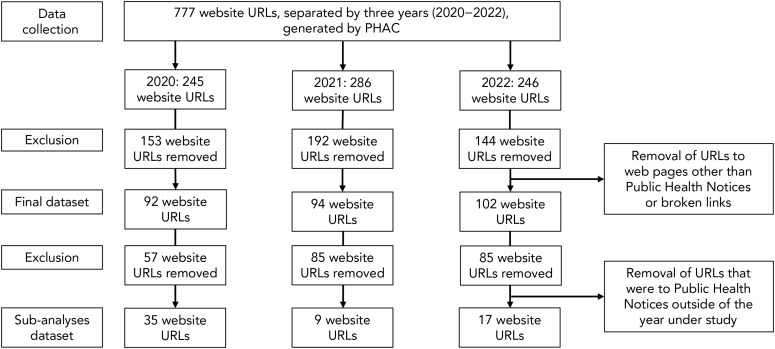
A step-by-step flowchart of URL selection criteria for analyses of data collection and filtering process for Public Health Notice URLs, 2020–2022 Abbreviations: PHAC, Public Health Agency of Canada; URL, Uniform Resource Locator

Web traffic and engagement data were obtained from PHAC’s Adobe Analytics system from January 1, 2020 to December 31, 2022 and filtered for inclusion (**Figure 1**). Several indicators were used to assess visitor behaviour, including page and screen metrics, geographic location, and device and traffic source metrics (**Table 1**). Data included number of visits, page views, page views per visit, average time on site, and outbound clicks for each PHN webpage Uniform Resource Locator (URL). Information associated with pages other than PHNs and broken links were removed. Visits to PHN webpages outside the study years (2020–2022) were initially included in the dataset but were removed and examined separately in a sub-analysis. The study period was chosen to reflect the most recent communication practices, focusing on PHNs published and updated within these years. Public Health Notices from outside this timeframe were not relevant, as the objective was to analyze traffic specific to PHNs published and updated during the study years. The number of PHN webpages does not reflect the unique number of multi-jurisdictional enteric illness outbreaks. The number of PHN webpages is higher because each PHN is available in English and French, and periodic updates to PHNs result in new versions stored as separate URLs. In aggregate, geographic location, visits per device type, and traffic source metrics were provided for all PHN webpages by year. Traffic source metrics were in the form of referring URL domains. Each URL domain was examined and grouped into four common traffic sources: direct, search, social media, and referral. Each common traffic source, except for direct, was further categorized into appropriate subcategories; for example, social media was grouped into type: Facebook, X (formerly Twitter), Reddit, LinkedIn, and Instagram.

**Table 1:** Description of metrics (page and screen, device, geographic location, and traffic source)

Metric	Metric description
Page and screen metrics	Not applicable
Visits	Number of times a user first arrives on the site The visit ends when any of the following criteria are met: 30 minutes of inactivity; 12 hours of activity; 2,500 hits; or 100 hits in 100 seconds
Page views	Number of times a visitor views a page
Average page views/visit	Average number of page views per visit
Average time on site	Average length of time visitors spends per session in seconds
Outbound clicks	Number of times a link is used to enter another website
Geographic location	Country (and province/territory or state for Canada and the United States) from which the webpage visit originated
Device type	Device used to access the webpage (mobile, desktop, or unspecified) during a visit
Traffic source	Source from which a visitor accesses the webpage
Search	Access from a search engine (e.g., Bing, Yahoo, Google)
Direct	Access via a bookmark or direct Uniform Resource Locator (URL)
Social media	Access via social media websites (e.g., X [formerly Twitter], Facebook, LinkedIn)
Referrals	Access via links on an external website

### Statistical analysis

Descriptive statistics (mean, standard deviation, and maximum and minimum values) were calculated for all continuous variables (page and screen metrics). A sub-analysis of page and screen metrics was performed to include only those PHNs that were posted or updated within the study period. This sub-analysis ensures that only PHN webpages relevant to the study period were included, while PHN webpages from earlier years, which were expected to have low traffic and engagement due to their outdated content, were excluded. This filtering process helped to more accurately capture the traffic and engagement patterns of webpages relevant to the study period. The year in which outbreaks occurred was classified according to the start date. A three-year average and standard deviation were also calculated for these variables to account for variations across the study period. The top 5% of visits, referring to the webpages that ranked within the highest 5% of total visits, were analyzed to identify trends in traffic and outbreak characteristics associated with heightened activity. The top 5% of outbound link clicks, representing the links that received the highest frequency of interactions, were examined to determine the types of information that users sought after visiting a PHN webpage. For geographic location, device type, and traffic source metrics, proportional frequencies were calculated for each respective year, in addition to a three-year average. An analysis of variance (ANOVA) test was performed to detect significant differences (α=0.05) between the annual average page and screen metrics and year. A chi-square test of independence was performed to detect significant differences (α=0.05) between years and geographic location, device type, and traffic source metrics. Data were tabulated and visualized using R Studio version base packages ((22)) and readxl ((23)), dplyr ((24)), openxlsx ((25)), rnaturalearth ((26)), rnaturalearthdata ((27)), rnaturalearthhires ((28)), tidyverse ((29)), cowplot ((30)), ggspatial ((31)), sf ((32)), png ((33)), grid ((34)), gridExtra ((35)), raster ((36)) and devtools packages ((37)).

### Ethics

Institutional ethics approval was not required as the data provided by PHAC were anonymized and derived from publicly accessible PHN webpages that do not require a login, password, or other restrictions to access.

## Results

### Page and screen metrics

A decreasing trend in page views and visits from 2020 to 2022 was observed (**Table 2**). Page views averaged 2,729±16,685 views while visits averaged 2,490±15,201 visits over the three years. In contrast, average session duration showed an increasing trend, rising from 148.9±108.0 seconds in 2020 to 183.8±157.1 seconds in 2022. While page views and visits trended downward, no statistically significant differences were found in page views (F[2,285]=0.49, *p*=0.61), visits (F[2,285]=0.49, *p*=0.61), session duration (F[2,285]=2.01, *p*=0.14), or page views per visit (F[2,285]=0.03, *p*=0.97) over time.

**Table 2:** Descriptive statistics of page and screen metrics for Public Health Notice webpages on the Government of Canada’s website, 2020–2022

Variable	2020^a^				2021^b^			2022^c^		
	Mean	Standard deviation	Range		Mean	Standard deviation	Range	Mean	Standard deviation	Range
Average page views	4,122	26,914		1–257,256	2,346	9,500	1–74,415	1,826	7,251	1–55,747
Average visits	3,758	24,448		1–233,603	2,139	8,778	1–70,165	1,670	6,697	1–52,055
Average page views per visit	1.1	0.2		1–2	1.1	0.1	1–1.5	1.1	0.2	1–2
Average session duration(s)	148.9	108.0		0–575	161.1	92.9	0–401.6	183.8	157.1	0–1,054.5

^a^ n=92 Public Health Notice webpages

^b^ n=94 Public Health Notice webpages

^c^ n=102 Public Health Notice webpages

The top 5% of visits averaged 36,637±55,515 visits. The top 5% of visits were associated with *Salmonella*, Hepatitis A and norovirus outbreaks involving produce, shellfish, and eggs. The top 5% of visits were for outbreak sizes ranging from three to 515 cases over the study period (**Table 3**).

**Table 3:** Top 5% of visits to Public Health Notice webpages and their associated pathogen, implicated source and outbreak size, 2020–2022

Year	Number of visits	Pathogen	Implicated source	Outbreak size (number of cases)
2020	233,603	*Salmonella*	Produce	515
	19,560	*Vibrio parahaemolyticus*	Shellfish	23
	16,450	*Salmonella*	Produce	57
	12,123^a^	*Salmonella*	Produce	515
	9,093	*Cyclospora*	Produce	370
2021	70,165	*Salmonella*	Eggs	70
	40,066	Hepatitis A	Produce	3
	22,003	*Salmonella*	Produce	118
	20,020^a^	*Salmonella*	Eggs	70
	9,687^a^	Hepatitis A	Produce	3
2022	52,055	Hepatitis A	Produce	10
	36,049	Norovirus	Shellfish	339
	24,104	Norovirus	Shellfish	60
	7,995	*Salmonella*	Produce	118
	7,919^a^	*Salmonella*	Eggs	70

^a^ Public Health Notice webpages communicated in French

The top 5% of outbound clicks consistently directed visitors to recall and safety alerts on the Government of Canada’s website (n=983). Additionally, in 2020, these clicks led users to the CDC outbreak information (n=420) and travel health notices on the Government of Canada’s website (n=412). In 2021, most outbound clicks were to recall and safety alerts (n=363) but also included contact information on the Government of Canada’s website (n=82). In 2022, clicks were to specific recall dates and safety alerts on the Government of Canada’s website (n=1,716).

### Page and screen metrics (sub-analysis)

A decreasing trend in page views and visits from 2020 to 2022 was observed. Across the study period, page views averaged 11,002±35,087 views while visits averaged 10,057±31,954 visits. In contrast, average session duration showed an increasing trend, rising from 94.6±77.3 seconds in 2020 to 184.2±238.7 seconds in 2022 (**Table 4**). While page views and visits trended downward, results indicated no statistically significant differences in page views (F[2,58]=0.17, *p*=0.84), visits (F[2,58]=0.17, *p*=0.84), session duration (F[2,58]=2.39, *p*=0.10), or page views per visit (F[2,58]=0.13, *p*=0.88) over time.

**Table 4:** A sub-analysis and descriptive statistics for Public Health Notices^a^ and screen metrics on the Government of Canada’s website, 2020–2022

Variable	2020^b^			2021^c^			2022^d^		
	Mean	Standard deviation	Range	Mean	Standard deviation	Range	Mean	Standard deviation	Range
Average page views	10,475	43,260	1–257,256	17,148	26,415	1–74,415	8,832	16,249	1–55,747
Average visits	9,559	39,289	1–233,603	15,713	24,501	1–70,165	8,087	15,028	1–52,055
Average page views/visit	1.1	0.2	1–2	1.1	0.2	1–1.5	1.2	0.2	1–2
Average session duration(s)	94.6	77.3	0–265.3	101.8	75.6	0–194.0	184.2	238.7	0–1,032

^a^ Public Health Notices posted and updated in their respective calendar year

^b^ n=35 Public Health Notice webpages

^c^ n=9 Public Health Notice webpages

^d^ n=17 Public Health Notice webpages

The top 5% of visits averaged 93,846±95,495 visits. Common pathogens included *Salmonella*, *Vibrio parahaemolyticus* and Hepatitis A, with produce, shellfish and eggs being frequently implicated sources. Outbreak sizes ranged from 10 to 515 cases (**Table 5**).

**Table 5:** A sub-analysis for the top 5% of visits to Public Health Notices^a^ webpages and their associated pathogen, implicated source and outbreak size, 2020–2022

Year	Number of visits	Pathogen	Implicated source	Outbreak size (number of cases)
2020	233,603	*Salmonella*	Produce	515
	19,560	*Vibrio parahaemolyticus*	Shellfish	23
2021	70,165	*Salmonella*	Eggs	70
2022	52,055	Hepatitis A	Produce	10

^a^ Public Health Notices posted and updated in their respective calendar year

### Geographic location metric

**Figure 2:**
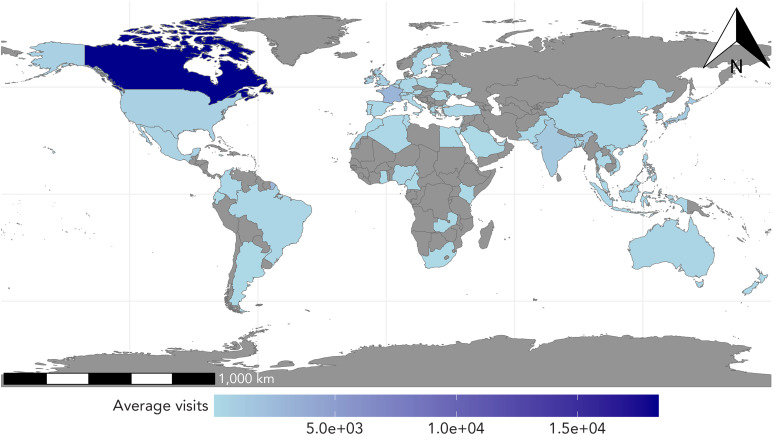
A choropleth map visualizing the three-year average visits from web traffic origins to Public Health Notice webpages on the Government of Canada’s website for the World^a^, 2020–2022 ^a^ Areas shaded grey represent areas from which no visits were recorded

Most PHN webpage visits originated from Canada, representing 89.0%±4.2% across the study period. A smaller percentage of visits came from the US (5.5%±1.7%). To a lesser extent, visitors accessed PHNs from outside of North America, including Asia, Africa, South America, Europe, and Oceania (0.1%±0.4%) (**Figure 2**). Geographic visits significantly differed by country and year (X^2^=30,606, degrees of freedom=116, *p*<0.001).

**Figure 3:**
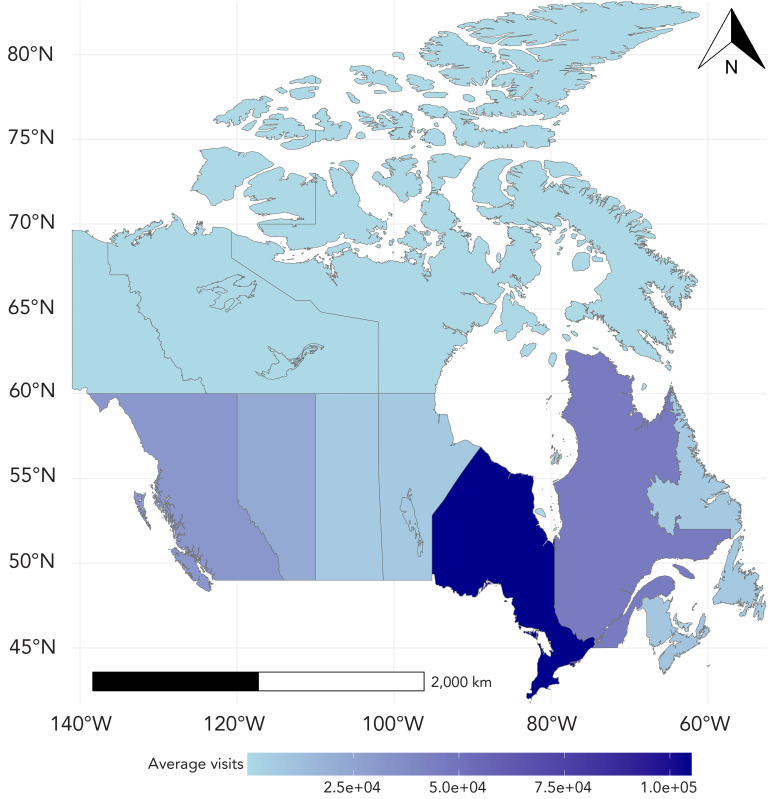
A choropleth map visualizing the three-year average visits from web traffic origins to Public Health Notice webpages on the Government of Canada’s website for Canada, 2020–2022

In Canada, Ontario had the highest proportion of visits each year, declining from 46.5% in 2020 to 40.1% in 2022, while Québec’s share increased from 14.7% to 21.8% over the same period. The territories, including Yukon, Northwest Territories and Nunavut, consistently accounted for less than 1% of visits throughout the study period. These percentages represent the proportion of visits to the webpages originating from different provinces and territories in Canada (**Table 6**, **Figure 3**). Geographic visits significantly differed by Canadian provinces and territories between years (X^2^=38,433, degrees of freedom=26, *p*<0.001).

**Table 6:** Number and proportion of visits from the United States, Canada, and Canadian provinces/territories to Public Health Notice webpages, 2020–2022

Country/province/territory	Number of visits to PHN webpages (% of total)		
	2020	2021	2022
United States	19,386 (5.4%)	8,441 (3.8%)	17,232 (7.1%)
Canada	328,436 (91.4%)	202,071 (90.6%)	202,078 (83.8%)
Ontario	152,796 (46.5%)	81,067 (40.1%)	81,067 (40.1%)
Québec	48,402 (14.7%)	43,969 (21.8%)	43,969 (21.8%)
British Columbia	58,166 (17.7%)	17,964 (8.9%)	17,964 (8.9%)
Alberta	34,565 (10.5%)	16,066 (8.0%)	16,066 (8.0%)
Nova Scotia	6,839 (2.1%)	10,763 (5.3%)	10,763 (5.3%)
New Brunswick	5,243 (1.6%)	9,993 (5.0%)	9,993 (5.0%)
Newfoundland and Labrador	2,909 (0.9%)	9,402 (4.7%)	9,402 (4.7%)
Manitoba	9,357 (2.9%)	5,843 (2.9%)	5,843 (2.9%)
Saskatchewan	8,105 (2.47%)	5,713 (2.8%)	5,713 (2.8%)
Prince Edward Island	1,619 (0.5%)	1,019 (0.5%)	1,019 (0.5%)
Yukon	223 (0.1%)	130 (0.1%)	130 (0.1%)
Northwest Territories	184 (0.1%)	116 (0.1%)	116 (0.1%)
Nunavut	28 (0.0%)	26 (0.0%)	26 (0.0%)
Unspecified	0 (0.0%)	0 (0.0%)	7 (0.0%)

Abbreviation: PHN, Public Health Notice

**Figure 4:**
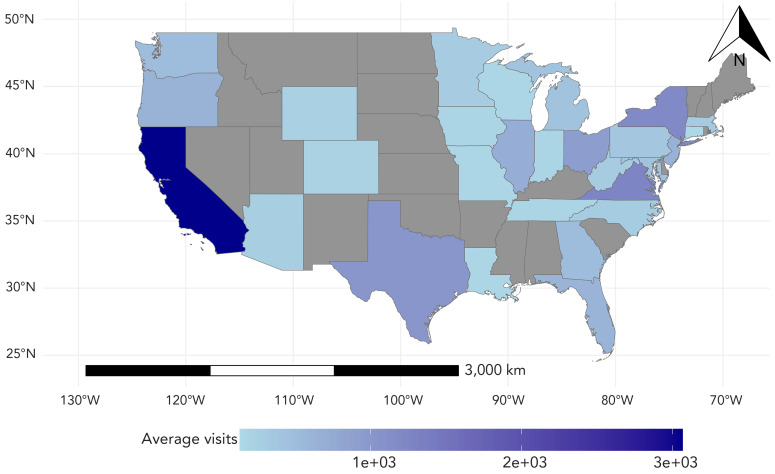
A choropleth map visualizing the three-year average visits from web traffic origins to Public Health Notice webpages on the Government of Canada’s website for United States^a^, 2020–2022 ^a^ Areas shaded grey represent areas from which no visits were recorded

In the US, California consistently had the highest proportion of visits each year, ranging from 22.9% in 2020 to 16.3% in 2022. The second largest contributor to visits varied, with Texas in 2020 (7.7%), Virginia in 2021 (12.3%), and New York in 2022 (10.6%) (**Figure 4**). Geographic visits significantly differed by state and year (X^2^=5,083.5, degrees of freedom=54, *p*<0.001).

### Device and browser metrics

The largest number of visits came from mobile devices (74.6%±3.3%), on average, over the three-year period. Although desktop visits were fewer, their proportion increased from 21.7% in 2020 to 29.5% in 2022 (**Table 7**). Device type significantly differed between years (X^2^=9,524.8, degrees of freedom=4, *p*<0.001).

**Table 7:** Device type trends (mobile, desktop, and unspecified) while accessing Public Health Notice webpages on the Government of Canada’s website over three years, 2020–2022

Device type	n (%)		
	2020	2021	2022
Mobile	282,761 (77.0%)	167,460 (74.3%)	133,958 (70.5%)
Desktop	79,541 (21.7%)	58,060 (25.7%)	56,002 (29.5%)
Unspecified	5,002 (1.4%)	13 (0%)	10 (0%)

### Traffic source metrics

**Figure 5:**
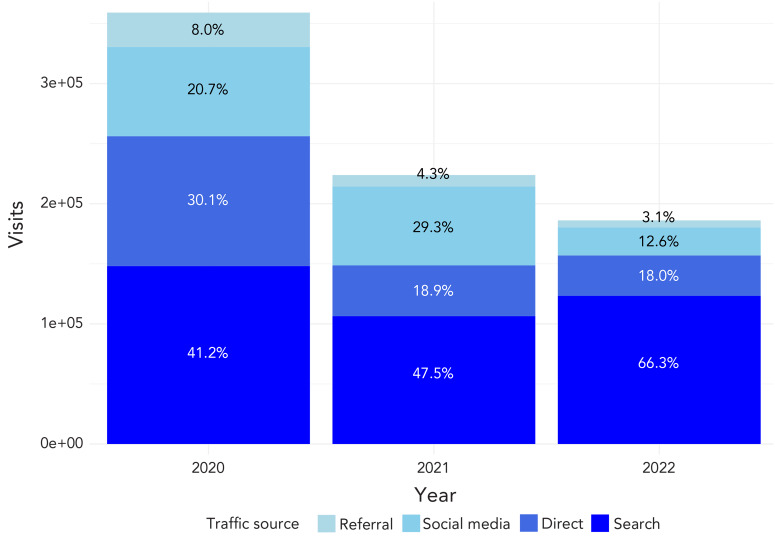
Traffic source distribution (search, direct, social media and referral) trends to Public Health Notice webpages on the Government of Canada’s website, 2020–2022

Search traffic remained the primary source of PHN visits (49.1%±13.0%), on average, over the three years, with Google dominating search traffic (97.0%±0.4%). Direct traffic was the second largest contributor (23.9%±6.7%), on average, showing a decline from 30.1% in 2020 to 18.0% in 2022. Social media, the third largest source (21.2%±8.4%), on average, peaked at 29.3% in 2021 before decreasing to 12.6% in 2022, with Facebook as the leading platform (73.1%±2.0%), followed by X (formerly Twitter) (25.1%±1.5%). Referral traffic, the smallest source, declined from 8.0% in 2020 to 3.1% in 2022. Government agencies (38.2%±6.4%) and news websites (40.7%±11.1%) were the main contributors to referral traffic over the period (**Figure 5**). Traffic sources significantly differed over the years (X^2^=46,097, degrees of freedom=6, *p*<0.001), with search traffic increasing while direct, social media, and referral sources declined.

## Discussion

### Principal findings

This study used descriptive analysis to quantify and describe website traffic and engagement data to PHN webpages on the Government of Canada’s website from 2020 to 2022 inclusive. Page and screen metrics, including visits and page views, showed decreasing traffic and engagement during this period, although average session duration remained consistently ideal (two to three minutes or longer). Most visits to PHN webpages originated from Canada, with a small proportion from international visitors. Despite fluctuations over the three years of the study, mobile devices constituted the majority of visits to PHN webpages overall, with a slight decline in mobile use and increase in desktop use. Google search emerged as the primary contributor to PHN webpage traffic, followed by direct, Facebook social media, and referral sources, in decreasing order.

### Page and screen metrics

The page and screen metrics (page views, visits, page views per visit, and average session duration) helped to understand user experience and navigation to aid in content creation ((38)). Public Health Notice webpages in 2020 demonstrated the largest engagement, marked by increased page views and visits, likely due to the relatively large number of outbreaks ((11)) and the scope of these outbreaks. From 2020 to 2022, the largest outbreaks regarding the number of cases occurred in 2020. For example, a *Salmonella* outbreak related to red onions was international in scope and resulted in 515 Canadian cases. In 2022, despite experiencing a higher number of outbreaks compared to 2021, there was a notable decrease in engagement, marking the lowest level of engagement observed throughout the study period. Given that the top 5% of visits over the study period varied by outbreak size, pathogen, and implicated sources, conclusive relationships to explain why some PHN webpages experienced higher visits cannot be determined.

Decreased PHN traffic may correlate with the COVID-19 pandemic, influencing Canadians’ online behaviours ((39)). During the pandemic, there was an increase in Internet-related activities ((40)), helping to explain the PHN webpages traffic peak in 2020; however, awareness of communicable diseases tends to wane during the resolution of a pandemic ((41)), which may explain why PHN webpage traffic decreased over the study period. Additionally, amid the COVID-19 pandemic, governments were placed in an unprecedented position—challenged by an ever-evolving situation to curb the spread of disease ((42)). During this time, Canadians’ trust in the government was challenged ((43)). A previous study surveying adults from the US found that government website use was positively associated with website satisfaction, which included accessible and complete information, and influenced citizen trust in government ((44)). Additionally, citizen trust in government influenced website satisfaction and, consequently, government website use ((44)). Similarly, in Canada, distrust in the government throughout the study period may have contributed to decreased access to PHN webpages. As the ease of access to government information is crucial to fostering transparency ((45)), the decline in trust may reflect a broader perception that information from public health authorities, including PHN webpages, was not readily accessible or adequately transparent ((46)). Ensuring information is readily accessible and available to the public through channels and formats tailored to their needs helps foster transparency ((47)), and when governments fail to meet these standards, it can erode trust.

During the pandemic, many Canadians used social media as a source of information ((48)). While social media can increase the perceived transparency of government actions, simply posting on social media is not enough to build trust ((46)). Increasing interactions with government information and ensuring information transparency are necessary ((46)). Ultimately, using social media to improve transparency can positively influence trust, which, in turn, can enhance PHN webpage use ((44)). Alternatively, the decreased access to PHN webpages may have been linked to the reduced reporting of communicable diseases to public health officials early in the COVID-19 pandemic (2019–2020) ((49)), including enteric illnesses in Canada ((50)). The cause of decreased enteric illness reporting is complex, and whether there was a true decrease in enteric illnesses due to eating habits or lockdown measures rather than a lack of reporting due to healthcare-seeking behaviour change is unclear ((50)). Nonetheless, the reduction in enteric illness reporting likely contributed to the decline in multi-jurisdictional enteric illness outbreaks and updates; therefore, the decrease in PHN webpage traffic could be explained by a reduced need to access the PHN website due to a lower frequency of multi-jurisdictional enteric illness outbreaks and updates.

In alignment with the general guideline recognizing that session lengths can vary depending on factors such as website type, industry, and user behaviour, PHAC webpages maintained an ideal average session duration of two to three minutes or longer, which may indicate the webpage captures users’ attention and keeps them engaged ((51)). Webpage factors that contribute to average session duration include webpage design, layout, and format ((52)). A content analysis of PHNs found that their design, layout, and format mostly aligned with the CDC’s Clear Communication Index best practices, which may have contributed to the webpages’ ideal average session length ((53)). Alternatively, the COVID-19 pandemic caused people to excessively search for health information on the Internet to relieve their health anxiety ((54)), which may explain the sustained engagement among users who did access PHN webpages. Despite declining engagement, as shown by decreasing page views and visits, those who access PHN webpages appear more engaged, as evidenced by the ideal average session duration.

### Geographic location metric

The geographic location metric offers insights into the demographic profile of website visitors by providing a broad overview of audience distribution, which can be used to help improve user experience for individuals of varying geographic areas ((38)). This includes the number of website visits from each country and a breakdown per region within North America. Considering PHN’s emphasis on sharing information about multi-jurisdictional enteric illness outbreaks in Canada, a high number of webpage visitors from Canada is expected. Most multi-jurisdictional enteric illness outbreaks during the study period occurred in Central Canada, the West Coast, and the Prairie provinces, which may explain the high number of webpage visitors from these regions. Ensuring those in affected areas are accessing the information they need has proven to have a positive effect on curbing the spread of communicable diseases ((55)).

Despite the localized nature of the outbreaks in Canada and the US, there was a small international viewership. Due to many cross-border outbreaks, website visits from Americans are expected. For example, in 2020 alone, there was a 515-case outbreak linked to red onions from the US ((56)) and a 57-case outbreak linked to peaches from the US ((57)). Visitors may have been redirected to PHN webpages via the CDC website, which routinely posts about foodborne outbreaks and links directly to PHAC’s PHNs when an outbreak has cross-border relevance. The small international presence could be explained by Canadian users accessing PHN webpages using virtual private networks to preserve their privacy ((58)) or Canadians travelling or living abroad who maintain their usual online activities. Alternatively, the small international presence (outside of both Canada and the US) may reflect travellers to Canada seeking information to protect themselves from foodborne illness ((59)) or the impact of public health digital surveillance abroad ((60)). Acknowledging PHAC’s guiding principles as part of the Government of Canada to prioritize communication with Canadians ((61)), it is not recommended to direct efforts to communicate with individuals residing in other countries.

### Device and browser metrics

Despite increased mobile device use during the COVID-19 pandemic ((62)), PHN webpages experienced a decline in mobile visits and an increase in desktop visits from 2020 to 2022, though mobile devices still constituted the majority. The COVID-19 pandemic and response caused a shift in digital use, thrusting both individuals and organizations online ((63)). In general, for those who used multiple devices to access the Internet, their choice of device varied depending on several factors, including the complexity and length of the task, time of day, or whether they were at home or work ((64)). While younger users tend to favour mobile devices to access information on the Internet, older adults prefer the computer ((65)). Additionally, the most noticeable shift in Internet use was amongst Canadians aged 50 to 64 years and aged 65 years or older ((63)). The observed increase in desktop use to access PHN webpages is consistent with shifts in digital behaviour seen during the COVID-19 pandemic response, likely influenced by factors such as stay-at-home orders, changes in work environment, the complexity of PHN webpages, and user demographics.

Although desktop use increased during this period, mobile devices still constituted the majority of visits to PHN webpages, highlighting the importance of optimizing webpages for seamless user interaction on mobile devices. Mobile optimization involves designing webpages specifically for mobile devices or adapting desktop layouts for mobile compatibility without altering the original structure ((66)). For tasks requiring cognitive processing, for example, reading information about multi-jurisdictional enteric illness outbreaks in Canada, younger adult users prefer simple, intuitive designs such as single-page scrolling (sliding vertically) or multi-page navigation (tapping through different pages) rather than zooming (two fingers moving in opposite directions) to accomplish tasks efficiently ((67)). However, these may pose usability challenges for older adults ((68)). Further, younger adults prefer a homepage with a thumbnail design that allows for quick content recognition and minimizes reliance on text-based titles ((67)). While older adults experience usability challenges while navigating different webpages, a thumbnail design was found to promote understanding, navigation, and interaction with online content ((68)). Currently, PHN webpages feature single-page scrolling and do not incorporate a thumbnail design, which could enhance useability and navigation. Understanding the demographic differences in device use and preferences can help to tailor PHN webpage design and optimization strategies to enhance user experience.

### Traffic source metrics

Traffic source metrics are used to track and analyze the source of traffic or channels visitors use to access a website, which can help understand how users discover and arrive at websites, aiding in marketing efforts ((38)). In agreement with literature emphasizing the importance of Google searches in driving website traffic ((69)), PHN webpages relied on Google searches as the primary traffic source. Search engine optimization is the practice of improving webpages to enhance their ranking in search results ((70)). To be specific, search engines use algorithms to match searched keywords with website content. The closer the match between the content and search queries, the higher the number of website visits. Search engine optimization can positively influence the usability ((71)) and the accessibility of websites ((72)); however, this study did not collect and analyze search terms to provide insight into search engine optimization.

Direct traffic represented the second largest channel for visitors, indicating a level of awareness or affinity for PHN pages ((73)). Visitors from direct channels are typically loyal and engaged users of the website ((73)), highlighting the affinity to and demand for PHN webpages.

Social media, particularly Facebook, emerged as the third largest channel. In Canada, Facebook is the largest social media network with an estimated 29.44 million monthly active users ((74)) and is popular among multiple demographic groups ((75)). This widespread use may contribute to increased PHN webpage traffic compared to other social media platforms. Previous research found a positive correlation between followership and predicted website traffic ((76)); however, our results contradict these findings. In November 2023, despite PHAC’s X followership (n=450,000) exceeding Facebook’s followership (n=433,000), the latter dominated as a social media traffic source. The COVID-19 pandemic spurred the use of social media as a crucial communication tool ((48)), with more people using Facebook than X for health information during the COVID-19 lockdown ((77)). Additionally, our previous study examining PHAC’s social media posts during multi-jurisdictional enteric illness outbreaks found that engagement was higher among Facebook posts compared to X, which could explain why Facebook remains the predominant social media traffic source ((78)). While PHAC has used X to disseminate information during multi-jurisdictional enteric illness outbreaks ((61)) and often provides links to corresponding PHNs ((78)), X contributed only about a quarter of social media search traffic. Given that X is predominantly used by younger adults ((79)) for health information ((77)), tailoring content to their preferences and needs could enhance X’s traffic volume to PHN webpages.

Overall, social media remains an underused traffic source, despite efforts during multi-jurisdictional enteric illness outbreaks to communicate through this channel and its importance in health information dissemination ((80)). A previous content analysis found that PHAC’s social media posts during multi-jurisdictional enteric illness outbreaks almost always used links; however, the study did not assess whether these links were incorporated according to best practices for clear links and to bolster engagement ((78)). The PHAC’s past social media posts on Facebook included phrases like “For more info” ((81)), “Additional details on recalled products, the outbreak investigation, and the latest food safety advice are here” ((82)) and “More information and health advice here” ((83)), followed by the URL to the corresponding PHN webpage. Similarly, posts on X (formerly Twitter) included phrases such as “more advice” ((84)), “more info” ((85)) and “get more information here” ((86)), also followed by a URL to the corresponding PHN webpage. However, according to the Web Content Accessibility Guidelines, links should be preceded by sufficient context for users to understand the purpose of the link without additional explanation ((87)). Phrases such as “more information” or “click here” are too vague and should be avoided ((88)). Additionally, URLs should not be included as link text, as they express little meaning to users ((89)). Instead, the text should be presented as descriptive and hyperlinked ((89)). Incorporating best practices, such as providing clear links to PHN webpages and encouraging engagement, could help bolster traffic and improve user interaction with PHAC’s PHN webpages.

Referral traffic remained a low contributor to website traffic, which may indicate low affiliate amplification and media coverage during multi-jurisdictional enteric illness outbreaks. In another study, it was found that although provincial and territorial partners had low amplification, the media communicated information during multi-jurisdictional enteric illness outbreaks, but at a low frequency ((90)). Furthermore, Canadian journalists are not legally mandated to cite their sources; they are only ethically encouraged to do so ((91)). This combination of low frequency of media coverage and the potential lack of citations to PHN webpages may contribute to the overall low levels of referral traffic. Given the importance that news media plays in communicating public health information ((92)), encouraging provincial, territorial, and media partners to share information and provide clear links to PHN webpages could help bolster referral traffic.

### Limitations

This descriptive analysis of website traffic and engagement on the Government of Canada’s website from 2020 to 2022 provided insights into visitor engagement as a proxy for awareness of PHN webpages; however, it has several limitations. First, the dataset used had deficiencies, particularly the absence of demographic information of users. This limitation hindered the ability to make associations between engagement metrics and demographic information, thereby precluding recommendations for content optimization tailored to specific demographics to enhance traffic and engagement. Second, aggregate data were used, as opposed to raw data, for the geographic location, device, and browser and traffic source metrics, posing limitations. Visits exclusively to PHN webpages within these metrics could not be confirmed, potentially including visits to other pages; however, visits to other pages are expected to comprise a small subset of the dataset. To contextualize social media traffic, PHAC’s recorded followership from November 2023 was used; however, this was from outside the study period of this article. Yet, the overall trend of greater followership on X compared to Facebook was not expected to change notably. Third, the absence of search terms restricted the ability to make recommendations regarding search engine optimization to enhance search as a traffic channel. Further, Adobe Analytics does not have available benchmarks to compare traffic and engagement metrics on PHN webpages to other government webpages. This made it difficult to effectively assess traffic to and engagement with PHN webpages. Additionally, the scope of the study was limited: inferences about whether visitors who accessed and engaged with the PHN webpages could understand and use the information to prevent enteric illnesses could not be made. Data was limited to a three-year period, which coincided with the COVID-19 pandemic (2020–2022), making it challenging to disentangle the pandemic’s impact and its influence on health information-seeking behaviours on government websites, particularly concerning multi-jurisdictional enteric illness outbreaks. Lastly, this analysis was descriptive and thus could only describe results without making associations or explaining relationships.

### Future research

This research quantified and described website traffic and engagement metrics as a proxy for audience awareness. Future research should explore relationships between traffic and engagement metrics, website design, and content to optimize engagement with information over a longer period for more reliable patterns of findings. Linking user engagement with website design and content can allow for designing more effective communications. Further, qualitative data should be collected to understand visitor traffic and engagement quantitative data, aiding in website optimization.

## Conclusion

As Internet use for accessing health-related information continues to rise, understanding website traffic and engagement becomes even more important. The PHAC uses PHNs posted on the Government of Canada’s website to disseminate outbreak investigation details and health protection information during multi-jurisdictional enteric illness outbreaks; however, traffic to and engagement with these webpages had previously been unexplored. While the average session duration remained optimal, there was decreasing engagement over the study period (2020–2022). Additionally, PHN webpages effectively attracted audiences from the affected provinces/territories. Visitors primarily used mobile devices to access PHN webpages, which are optimized for mobile web browsing, contributing to enhanced user experience. Google search emerged as the primary traffic source for accessing PHN webpages followed by direct, Facebook social media, and referral. Despite efforts used to communicate with individuals through social media, this traffic source remains underused. Website traffic and engagement analysis is a useful tool to assess and support visitor access to and engagement with PHN webpages. Continued monitoring and optimization efforts are essential to ensure effective communication of multi-jurisdictional enteric illness outbreaks to the public.
